# A nomogram prognostic model for early hepatocellular carcinoma with diabetes mellitus after primary liver resection based on the admission characteristics

**DOI:** 10.3389/fphar.2024.1360478

**Published:** 2024-02-15

**Authors:** Menghan Zhang, Qi Wang, Gongming Zhang, Guangming Li, Ronghua Jin, Huichun Xing

**Affiliations:** ^1^ Center of Liver Diseases Division 3, Beijing Ditan Hospital, Capital Medical University, Beijing, China; ^2^ Beijing Ditan Hospital, Capital Medical University, Beijing, China; ^3^ Department of General Surgery Center, Beijing YouAn Hospital, Beijing Institute of Hepatology, Capital Medical University, Beijing, China; ^4^ Changping Laboratory, Beijing, China; ^5^ Center of Liver Diseases Division 3, Beijing Ditan Hospital, Peking University, Beijing, China

**Keywords:** hepatocellular carcinoma, primary liver resection, diabetes mellitus, nomogram, overall survival

## Abstract

**Background:** Patients diagnosed with early-stage hepatocellular carcinoma (HCC) and diabetes mellitus (DM) are at a higher risk of experiencing complications and facing increased mortality rates. Hence, it is crucial to develop personalized clinical strategies for this particular subgroup upon their admission. The objective of this study is to determine the key prognostic factors in early HCC patients who received liver resection combined with DM and develop a practical personalized model for precise prediction of overall survival in these individuals.

**Method:** A total of 1496 patients diagnosed hepatitis B virus (HBV) - related liver cancer from Beijing You’an Hospital were retrospectively enrolled, spanning from 1 January 2014, to 31 December 2019, and ultimately, 622 eligible patients of hepatocellular carcinoma (HCC) patients with diabetes were included in this present investigation. A multivariate COX regression analysis was conducted to identify prognostic factors that are independent of each other and develop a nomogram. The performance of the nomogram was evaluated using various statistical measures such as the C-index, receiver operating characteristic (ROC) curves, calibration curves, and decision curve analysis (DCA) in both the training and validation groups. Survival rates were estimated using the Kaplan-Meier method.

**Results:** The study included a total of 622 early HCC patients who underwent liver resection combined with DM. Random Forrest model and Multivariate Cox regression analysis revealed that drinking, tumor number, monocyte-to-lymphocyte ratio, white blood cell count and international normalized ratio at admission were identified as independent prognostic factors for early HCC patients who underwent liver resection combined with DM. The nomogram demonstrated good predictive performance in the training and validation cohorts based on the C-index values of 0 .756 and 0 .739 respectively, as well as the area under the curve values for 3-, 5-, and 8-year overall survival (0.797, 0.807, 0.840, and 0.725, 0.791, 0.855). Calibration curves and decision curve analysis indicated high accuracy and net clinical benefit rates. Furthermore, the nomogram successfully stratified enrolled patients into low-risk and high-risk groups based on their risk of overall survival. The difference in overall survival between these two groups was statistically significant in both the training and validation cohorts (*p* < 0.0001 and *p* = 0.0064).

**Conclusion:** Our results indicate that the admission characteristics demonstrate a highly effective ability to predict the overall survival of early HCC patients who have undergone liver resection in combination with DM. The developed model has the potential to support healthcare professionals in making more informed initial clinical judgments for this particular subgroup of patients.

## Introduction

Hepatocellular carcinoma (HCC) stands as the sixth most prevalent cancer and ranks third among causes of cancer-related mortality (Sung et al., 2021; Waked et al., 2023). Therefore, HCC remains a significant global health concern that necessitates urgent attention. With the advancements in imaging technology, an increasing number of patients with early-stage HCC are being diagnosed, enabling potential treatment options such as liver resection, local ablation, or liver transplantation (Villanueva, 2019; Llovet et al., 2021). However, the long-term prognosis of HCC remains unfavorable and discouraging due to the limited rate of early diagnosis, the heterogeneity in oncology, and the elevated risk of early recurrence (Zhong et al., 2012a; Zhong et al., 2012b; Allemani et al., 2018).

Diabetes mellitus (DM) is a prevalent metabolic disorder characterized by chronic hyperglycemia resulting from impaired insulin secretion, insulin resistance, or a combination of both (DeFronzo et al., 2015). The presence of elevated insulin levels in the body inhibits the mitochondrial β-oxidation process of fatty acids, thereby promoting hepatocarcinogenesis through repeated stimulation of chronic inflammation and ultimately leading to the development of liver cancer. DM serves as an established risk factor for the rapid progression of non-alcoholic fatty liver disease (NAFLD) to cirrhosis or HCC (Mantovani and Targher, 2017; Targher et al., 2021). Moreover, there is a consistent body of evidence from epidemiological studies indicating that individuals who have been diagnosed with both chronic hepatitis B or C virus infection and diabetes mellitus are more likely to develop HCC (Veldt et al., 2008; Li et al., 2012; Dyal et al., 2016). However, there was a consistent absence of research on the assessment of autonomous prognostic factors and models to assist in clinical decision-making for early HCC patients who received liver resection combined with DM upon initial admission.

To bridge the existing research lacunae, the purpose of this study is to identify the significant prognostic factors in early HCC patients who underwent liver resection combined with DM. Furthermore, our objective is to develop and authenticate a feasible personalized framework that enables doctors to precisely anticipate the overall survival of these individuals. This would constitute an imperative stride towards the advancement of precision medicine.

## Material and methods

### Patients enrolled

The study enrolled a total of 1496 patients aged between 18 and 75 years who were admitted to Beijing You’an Hospital affiliated with Capital Medical University from 1 January 2014, to 31 December 2019 and regular follow-ups were conducted until 1 July 2023, and ultimately, 622 eligible patients were included in this present investigation. These individuals were identified as having early-stage hepatocellular carcinoma (HCC) through the utilization of alpha-fetoprotein (AFP), advanced imaging techniques, or histological analysis employing the diagnostic criteria endorsed by AASLD (Heimbach et al., 2018). Diabetes mellitus (DM) refers to patients with type 2 diabetes based on plasma glucose criteria following the guidelines (Classification and Diagnosis of Diabetes, 2022). The desired outcome of complete response was observed in all treated patients, with the target lesions exhibiting complete regression and the tumor markers remaining within the normal range for a minimum duration of 4 weeks (Choi et al., 2005).

Subjects meeting any of the following criteria were excluded: 1) presence of infection or inflammation during blood sampling; 2) patients with Barcelona Clinic Liver Cancer (BCLC) B- D; 3) secondary liver cancer; 4) accompanying other malignancies, such as different types of tumors or severe coagulopathy; 5) disorders in the coagulation function or severe illnesses impacting the heart, brain, lung, or kidney; and 6) other serious metabolic diseases and immune system diseases.

The patients were assigned randomly to a training set (N1 = 435) and a validation set (N2 = 187) in a ratio of 7:3, aiming to establish a more robust and dependable model. Following that, a comparison was made between the demographic characteristics, prognosis of patients, and laboratory data in both cohorts. The study protocol received approval from the Ethics Committee of Beijing You’an Hospital. Given its retrospective nature, written informed consent from patients was not required.

### Clinical data collection

The initial clinicopathological profiles of the individuals were gathered, encompassing age, sex, demographic factors (such as prior hypertension incidents, diabetes mellitus occurrences, antiviral therapy), laboratory measurements (including complete blood count analysis, hepatic function assessments, coagulation functionality evaluation, and hepatitis virus indicators), assessment of tumor burden (number and size of tumors), evaluation of tumor markers (alphafetoprotein [AFP] and des-γ-carboxyprothrombin [DCP]), etiology analysis.

### Follow-up

Patients were followed up within the first month after discharge, and subsequently at intervals of 3–6 months. The follow-up assessments included blood routine examination, liver function tests, alpha-fetoprotein (AFP) measurement, as well as CT/MRI scans. Regular follow-ups were conducted until 1 July 2023. Overall survival (OS) refers to the duration from randomization until death, encompassing patients who may be lost to follow-up or still alive at the time of evaluation, which are considered as censored cases (Lebwohl et al., 2009; Prinja et al., 2010).

### Statistical analysis

The mean ± standard deviation was used to represent the continuous variables, and group differences were assessed using Student’s t-test. The comparison of categorical variables, presented as frequency and percentages, was conducted using the Chi-square test. Survival curves were generated using the Kaplan-Meier method and compared using log-rank tests. Random survival forest analysis and multivariate Cox regression analyses were conducted to identify independent risk factors for overall survival in patients with HCC. Following this, a nomogram was created utilizing the Cox regression model for overall survival prediction. The prognostic nomogram was validated in an independent cohort of patients. Based on the nomogram scores, patients were categorized into low-risk, and high-risk groups with corresponding predicted rates of overall survival. Time-dependent receiver operating characteristic (ROC) curves were constructed to visualize the data, and the area under the ROC curve (AUC) was calculated to evaluate the prognostic significance of candidate factors. Calibration curves and the Hosmer-Lemeshow test were performed to assess the predictive accuracy of the nomogram model. The decision curve analysis (DCA) was utilized to demonstrate the clinical applicability of the nomogram model in both training and validation cohorts by quantifying its net benefits.

The statistical analyses were conducted using R software version 4.1.2 in this study. Two-tailed tests were employed for all analyses, with statistical significance defined as a *p*-value less than 0.05 (*p* < 0.05).

## Results

### Characteristics of patients

The study participants were recruited from Beijing You’an Hospital, Capital Medical University. A total of 1496 patients diagnosed hepatitis B virus (HBV) - related liver cancer between 2014 and 2019 underwent screening, and ultimately, 622 eligible patients were included in this present investigation, including 475 (76.4%) males and 147 (23.6%) females. Among the 622 patients, 260 (41.8%) had a history of smoking, and 203 (32.6%) had a history of drinking. There were 543 (87.3%) patients with cirrhosis. 466 (74.9%) were Child-Pugh class A, and 156 (25.1%) were Child-Pugh class B. Concerning the tumor characteristics, 69.3% of patients had a single tumor and 64.6% had a tumor size smaller than 3 cm, with most of the patients having BCLC stages A (69.9%). The demographic profile of the patients in the validation cohort is presented in Table 1. The baseline characteristics between the two cohorts did not exhibit any significant differences when compared to the historical data from the training cohort.

**TABLE 1:** Comparison of clinical data between training set and validation set.

Variables	Training set	Validation set	*p*-Value
	N = 435	N = 187	
WBC (10^9/L)	5.08 ± 2.16	5.07 ± 1.98	0.930
NLR	3.09 ± 2.50	3.27 ± 3.02	0.444
PLR	109.31 ± 58.19	107.32 ± 57.24	0.693
MLR	0.37 ± 0.21	0.37 ± 0.21	0.906
RBC (10^9/L)	4.14 ± 0.57	4.07 ± 0.62	0.214
Hb (g/L)	130.37 ± 18.33	128.39 ± 19.14	0.225
ALT (U/L)	30.72 ± 20.02	31.70 ± 19.05	0.570
AST (U/L)	32.09 ± 14.69	32.87 ± 16.11	0.554
TBIL (μ mol/L)	19.44 ± 10.01	18.76 ± 9.93	0.441
DBIL (μ mol/L)	6.46 ± 4.44	6.45 ± 4.82	0.981
Total protein (g/L)	65.387 ± 7.02	65.11 ± 6.42	0.646
ALB (g/L)	37.00 ± 4.66	37.19 ± 4.55	0.644
Globulin (g/L)	28.56 ± 5.37	27.93 ± 5.68	0.189
GGT (U/L)	62.75 ± 50.89	62.49 ± 48.35	0.951
ALP (U/L)	87.35 ± 32.73	83.87 ± 31.59	0.221
Prealbumin (g/L)	133.02 ± 56.75	136.31 ± 59.36	0.513
Bile acid (μ mol/L)	21.30 ± 26.71	21.36 ± 24.67	0.976
BUN (mmol/L)	5.06 ± 2.11	4.92 ± 1.85	0.443
Creatinine (μ mol/L)	67.74 ± 52.56	63.61 ± 14.46	0.290
Uric acid (μ mol/L)	281.92 ± 90.68	279.84 ± 86.38	0.791
Glucose (mmol/L)	5.84 ± 1.12	5.45 ± 1.29	0.519
Osmotic pressure (mmol/L)	297.57 ± 15.03	298.23 ± 4.73	0.552
Prothrombin time (s)	12.63 ± 1.58	12.47 ± 1.35	0.204
PTA (%)	85.68 ± 15.21	86.79 ± 14.39	0.397
PTR	1.12 ± 0.14	1.11 ± 0.11	0.479
INR	1.11 ± 0.13	1.11 ± 0.12	0.453
APTT (s)	33.66 ± 4.74	33.59 ± 4.19	0.859
Fibrinogen (g/L)	2.82 ± 0.94	2.79 ± 0.89	0.684
Thrombin time (s)	15.99 ± 2.26	15.80 ± 2.27	0.361
AFP (ng/mL)	334.5 ± 1231.1	209.38 ± 993.28	0.182
Gender (Male/Female)	338/97	137/50	0.258
Smoking (yes/no)	180/255	80/107	0.790
Drinking (yes/no)	143/292	60/127	0.926
Child-Pugh (A/B)	320/115	146/41	0.267
BCLC Stage (0/A)	123/312	64/123	0.153
Tumour number (single/multiple)	298/137	133/54	0.570
Tumour size (≤30mm/>30 mm)	274/161	128/59	0.201
Cirrhosis (yes/no)	381/54	162/25	0.793

Abbreviations: WBC, white blood cells; NLR, Neutrophil‐Lymphocyte Ratio; PLR, Platelet-to-Lymphoccyte Ratio; MLR, monocyte to lymphocyte; RBC, red blood cells; Hb, Hemoglobin; ALT, alanine aminotransferase; AST, aspartate aminotransferase; TBIL, total bilirubin; DBIL, direct bilirubin; ALB, albumin; GGT, glutamyl transferase; ALP, alkaline phosphatase; PTA, prothrombin time activity; INR, international normalized ratio; APTT, activated partial thromboplastin time; AFP, Alpha-fetoprotein; BCLC, barcelona clinic liver cancer.

### Screening for OS-related variables

**FIGURE 1:**
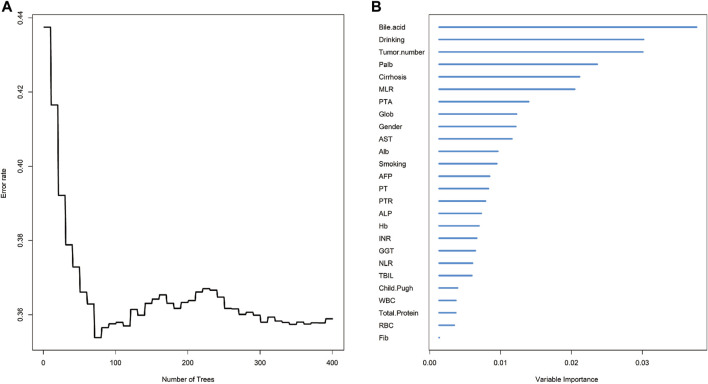
The overall survival analysis of HCC based on random survival forest. **(A)** Error rate of random survival forest; **(B)** out-of-bag variable importance ranking. Abbreviations: HCC: hepatocellular carcinoma; Palb: Prealbumin; MLR: Monocyte to Lymphocyte Ratio; Alb: Albumin; AST: Aspartate Transaminase; PTA: Prothrombin Activity; Glob: Globulin; PT: Prothrombin Time; ALP: Alkaline Phosphatase; AFP: Alpha Fetoprotein Hb: Hemoglobin; NLR: Neutrophil to Lymphocyte Ratio; GGT: Gamma-glutamyl Transpeptidase; INR: International Normalized Ratio; PTR: Prothrombin Time Ratio; WBC: White Blood Cell; TBIL: Total Bilirubin; Fib: Fibrinogen; RBC: Red Blood Cell.

Based on the aforementioned discussions, we ultimately selected the Random forest-Cox regression model as a predictive tool for assessing OS in patients with early-stage HCC and diabetes mellitus (DM) who underwent liver resection. The process of parameter debugging revealed that as the value of ntree increased to 400, there was a tendency for the model’s error rate to stabilize (Figure 1A) and the variables were prioritized based on the Variable Importance (VIMP) approach, as depicted in Figure 1B. We then performed a multivariate cox regression analysis, which showed that revealed that drinking (hazard ratio (HR) = 1.547, 95%CI: 1.013–2.369, *p* = 0.045), tumor number (HR = 1.362, 95%CI: 1.027–2.001, *p* = 0.116), monocyte-to-lymphocyte ratio (HR = 3.326, 95%CI: 1.074–6.297, *p* = 0.037), white blood cell count (HR = 0.008, 95%CI: 0.754–0.959, *p* = 0.008) and international normalized ratio (HR = 4.648, 95%CI: 3.321–6.411, *p* = 0.04) at admission were identified as independent prognostic factors for early HCC patients who underwent liver resection combined with DM (Table 2).

**TABLE 2:** Prognostic factors associated with OS by multivariate regression analysis in training set.

Variables	Multivariate	
	HR (95%CI)	*p*-value
Bile.acid	1.006 (0.997–1.015)	0.174
Drinking	1.547 (1.013–2.369)	0.045
Tumor.number	1.362 (1.027–2.001)	0.116
Palb	0.995 (0.989–1.315)	0.056
Cirrhosis	1.718 (0.399–2.293)	0.27
MLR	3.326 (1.074–6.297)	0.037
Alb	1.026 (0.931–1.131)	0.607
AST	1.996 (0.983–3.009)	0.579
PTA	0.969 (0.931–1.015)	0.14
Gender	0.873 (0.491–1.552)	0.643
Glob	1.056 (0.949–1.177)	0.318
PT	0.899 (0.503–1.606)	0.719
ALP	1.001 (0.994–1.008)	0.789
Smoking	1.982 (0.641–1.499)	0.927
AFP	1.124 (0.012–2.223)	0.557
Hb	1.015 (0.991–1.039)	0.226
NLR	1.019 (0.914–1.136)	0.738
GGT	1.003 (0.999–1.007)	0.156
INR	4.648 (3.321–6.411)	0.04
PTR	0.612 (0.009–1.384)	0.443
WBC	0.853 (0.754–0.959)	0.008
Total.protein	0.985 (0.891–1.078)	0.675
TBIL	1.999 (0.974–1.025)	0.961
Child-Pugh	0.932 (0.557–1.562)	0.791
Fib	1.115 (0.875–1.421)	0.377
RBC	0.795 (0.384–1.626)	0.522

Abbreviations: Palb, Prealbumin; MLR, monocyte to lymphocyte; ALB, albumin; AST, aspartate aminotransferase; PTA, prothrombin time activity; Glob, Globulin; PT, prothrombin time; ALP, alkaline phosphatase; AFP, Alpha-fetoprotein; Hb, Hemoglobin; NLR, Neutrophil‐Lymphocyte Ratio; GGT, glutamyl transferase, INR, international normalized ratio; PTR, prothrombin time ratio; TBIL, total bilirubin; Fib, Fibrinogen; RBC, red blood cell.

### Develop the nomogram

**FIGURE 2:**
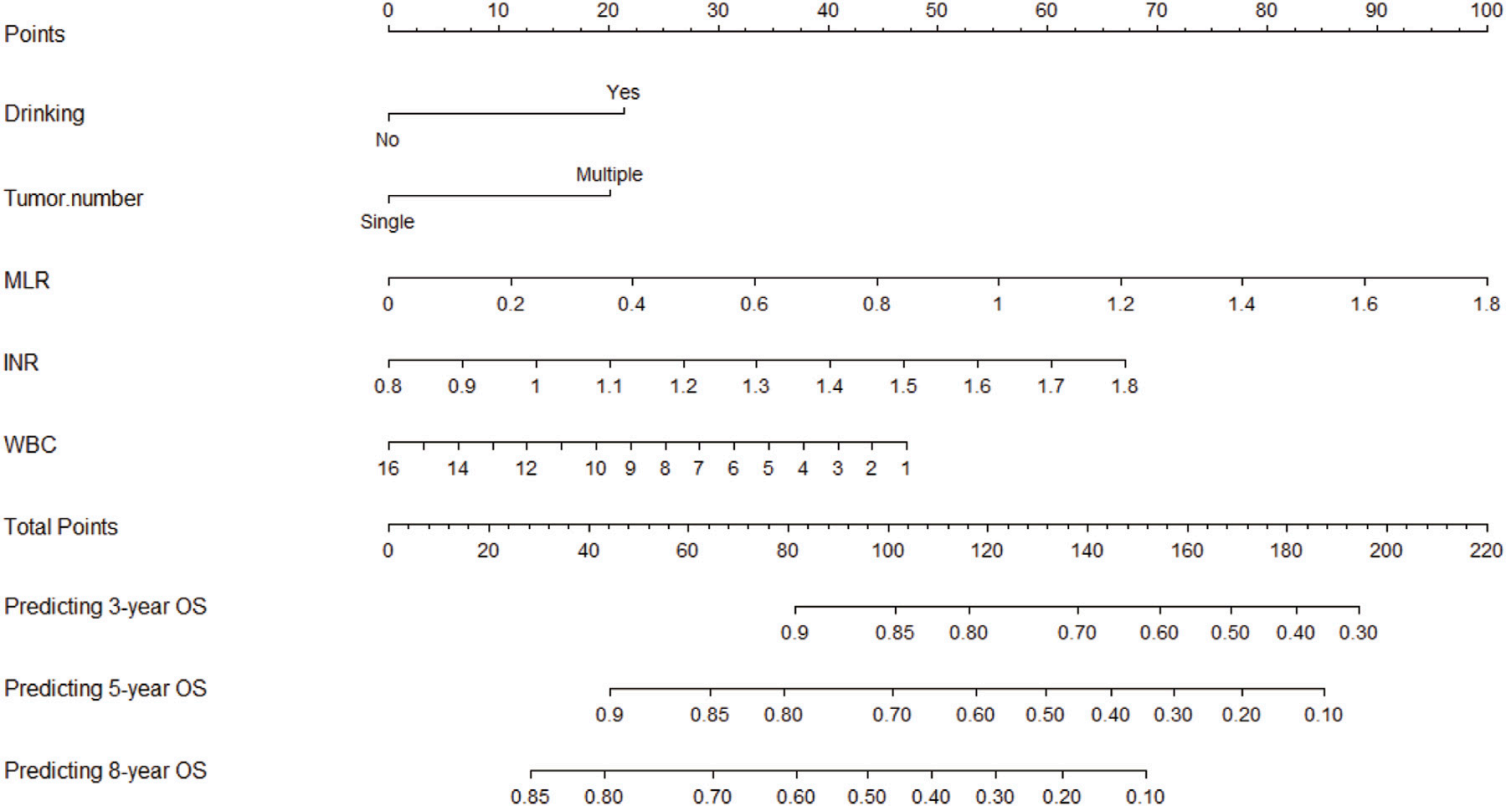
Nomogram used to predict time-related overall survival in patients with early-stage HCC. Abbreviations: HCC, hepatocellular carcinoma; MLR: Monocyte to Lymphocyte Ratio; NLR: Neutrophil to Lymphocyte Ratio; WBC: White Blood Cell; OS: Overall Survival.

To enhance its clinical applicability, we transformed the intricate mathematical model into a user-friendly nomogram (Figure 2). This necessitated the aggregation of scores assigned to each variable included in the model and drawing a vertical line at the total score intersecting with three lines representing predicted overall survival (OS) values. The corresponding intersection point values indicate an individual’s predicted rates of OS at 3, 5, and 8 years. For instance, an individual with a history of drinking and smoking, MLR of 0.8, INR of 1.2, and WBC count of 4 × 10^^^9 obtained a total score approximately equal to154, which corresponded to estimated OS rates around 67% at year-3, about 35% at year-5, and approximately 10% at year-8. It is evident that utilization of this nomogram offers enhanced convenience in clinical settings.

**FIGURE 3:**
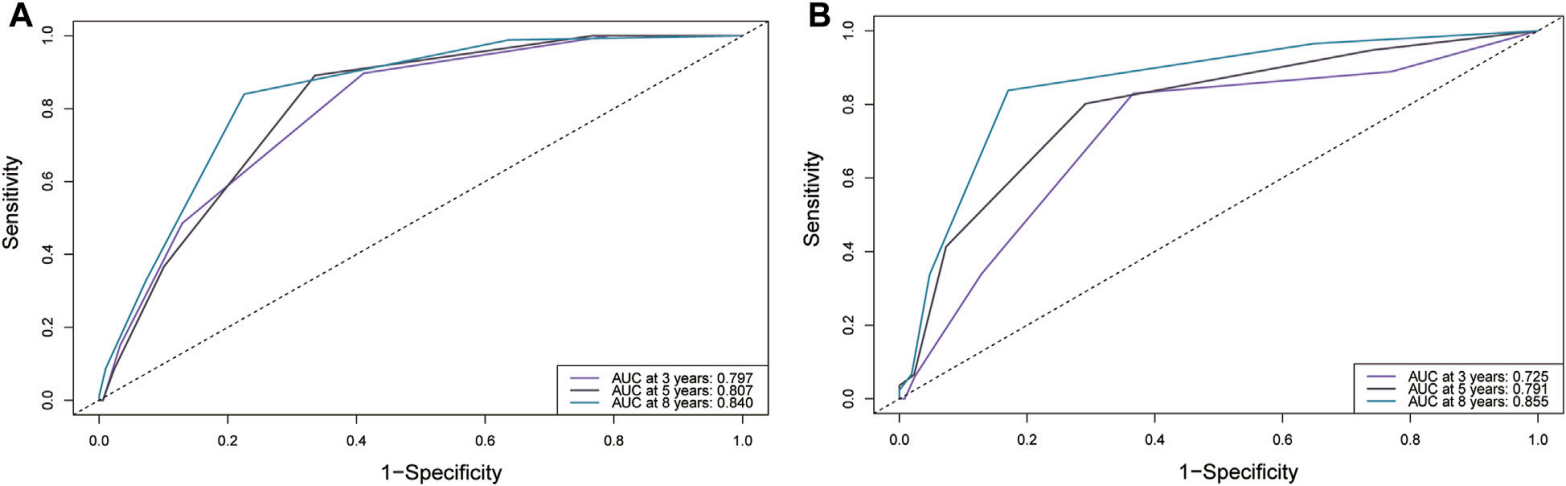
Comparison of the ROC curves of the original scoring system at different time points in the validation cohort. **(A)** training set; **(B)** validation set. Abbreviations: ROC: Receiver Operating Characteristics; AUC, Area under the Curve.

**FIGURE 4:**
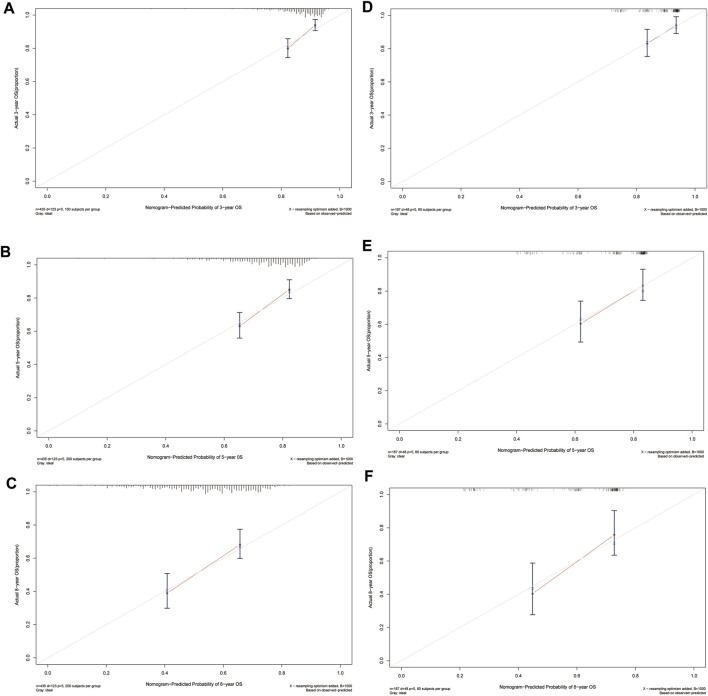
Calibration plots of predicted 3-, 5-, and 8-year OS based on Cox regression modeling in the training set and validation set. **(A–C)** training set; **(D–F)** validation set. Abbreviations: OS, overall survival.

**FIGURE 5:**
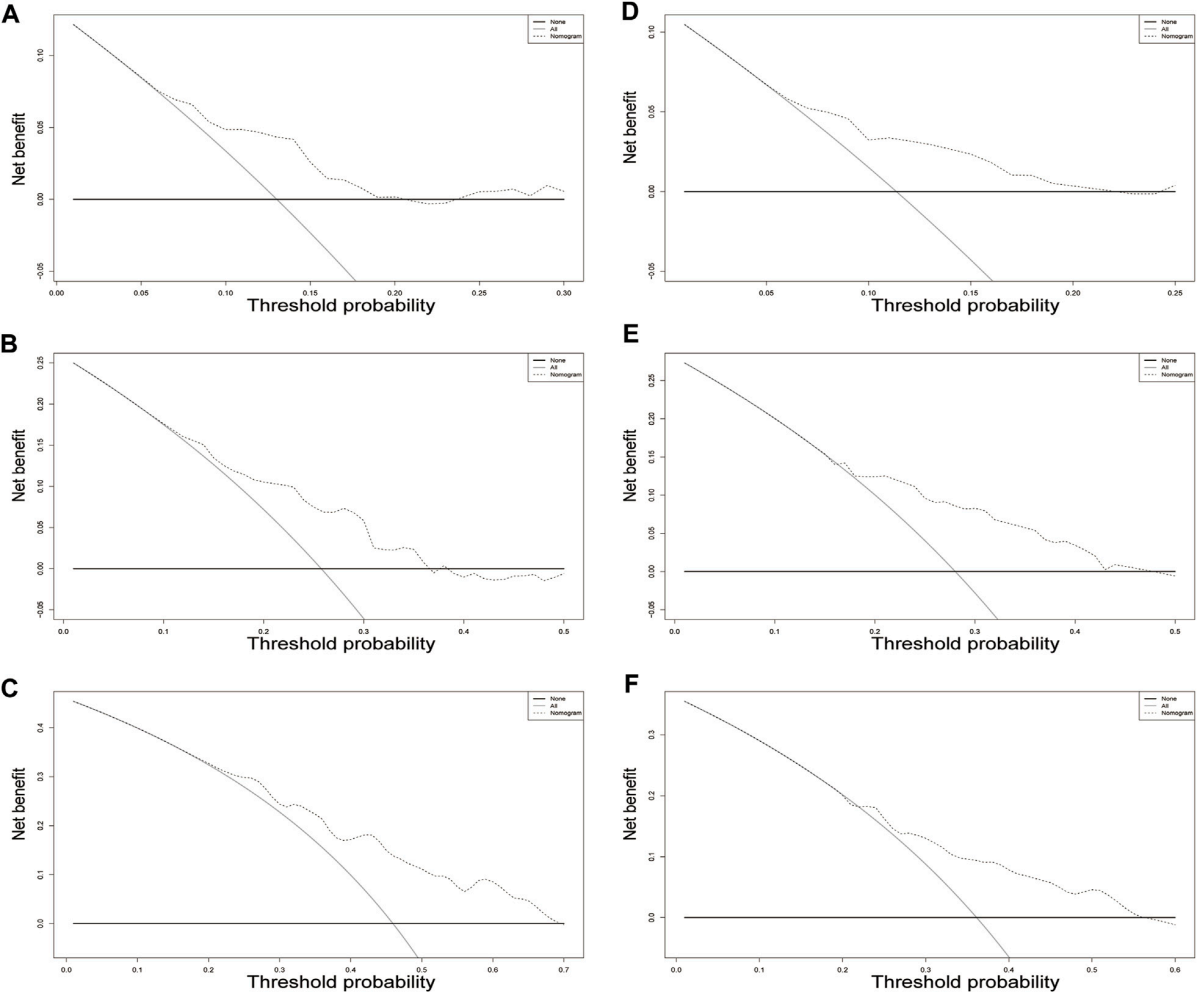
Decision curve analysis for overall survival in the training cohort. The *x*-axis indicates threshold probability, and the *y*-axis indicate the net benefit. Dashed lines: the net benefit of nomogram across a range of threshold probabilities. The solid red line: no patients relapse. The solid black line: all patients die or relapse. Decision curve analysis at different time points in the validation cohort. **(A–C)** training set; **(D–F)** validation set.

**FIGURE 6:**
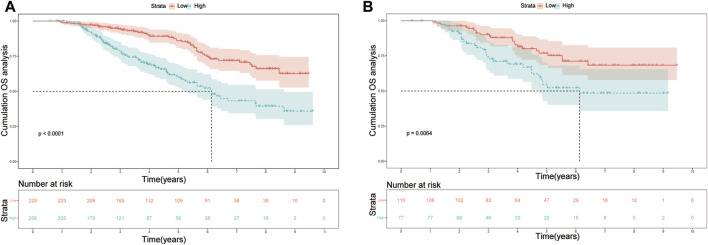
Kaplan-Meier plots of OS for the low-risk group and high-risk group in the **(A)** training set; **(B)** validation set. Abbreviations: OS, overall survival.

In the training cohort, the nomogram exhibited a robust predictive ability for OS, with a C-index of 0.756 (95% CI: 0.716–0.795). The time-dependent ROC curve further demonstrated exceptional discriminative performance, yielding AUCs of 0.797, 0.807, and 0.840 at 3-, 5-, and 8-year follow-up, respectively (Figure 3A). Furthermore, the calibration plot depicted satisfactory concordance between predicted probabilities and actual observations for OS at different time points (Figures 4A–C). Additionally, decision curve analysis illustrated favorable net benefits across various threshold probabilities when employing the nomogram in clinical practice (Figures 5A–C). By stratifying patients into low-risk and high-risk groups based on the nomogram predictions (Figure 6A), significant differences in OS were observed within the training cohort comprising a low-risk group (*n* = 229) and a high-risk group (*n* = 206) (*p* < 0.001).

### Validate the nomogram

To further validate the reliability of the nomogram, an internal verification was conducted. In the validation cohort, the C-index was 0.739 (95%CI: 0.662–0.815), and the time-independent AUCs for 3-, 5-, and 8-year were 0.725, 0.791, and 0.855 respectively (Figure 3B). The calibration curves also demonstrated a strong correspondence with observed data (Figures 4D–F). Moreover, decision curve analysis exhibited promising clinical applicability in predicting overall survival risk at different time points (Figures 5D–F). In addition, the validation cohort was divided into two groups based on risk level: a low-risk group (*n* = 110) and a high-risk group (*n* = 71). The results consistently demonstrated a significantly higher likelihood of overall survival in the high-risk group compared to the low-risk group. (*p* = 0.0064) (Figure 6B).

## Discussion

Currently, there is a lack of prognostic factors available for early HCC patients who have undergone liver resection combined with DM. This study fills this research gap by identifying various admission characteristics that demonstrate significant associations with overall survival in this specific subpopulation. Furthermore, a personalized prognostic nomogram has been created to assist in the clinical handling of these individuals during the initial assessment stage. Our comprehensive analyses confirm the outstanding ability of this predictive model in distinguishing and calibrating accurately, as well as its practicality in clinical settings.

In comparison to recent studies conducted on the general early HCC population who underwent liver resection, our current study has successfully validated several clinical indicators specifically in patients with DM. Interestingly, we observed that some inflammation factors (monocyte-to-lymphocyte ratio (MLR) and white blood cell count (WBC)) emerged as a robust prognostic determinant for overall survival in the early HCC patients who underwent liver resection combined with DM. It has been shown that insulin resistance (IR) and activation of the insulin receptor, as well as the insulin-like growth factor 1 (IGF-1) signaling pathways, play pivotal roles in both the initiation and progression of hepatocarcinogenesis in patients with DM (Smedile and Bugianesi, 2005; Hung et al., 2010; Kanda et al., 2020). Meanwhile, the exposure to IR elicits a diverse range of metabolic and molecular effects, which instigate inflammation, oxidative stress resulting in DNA damage, and activation of cellular pathways involved in cellular growth and proliferation. These cumulative processes collectively contribute to the potential development of HCC (Warburg, 1956). These present findings may partially elucidate the underlying mechanism through which the inflammatory pathway significantly impacts the prognosis of early HCC patients with DM who have undergone liver resection. Furthermore, various clinical researches have consistently shown that DM patients face a 2.5-4-fold higher risk of developing HCC, regardless of the presence of cirrhosis or the etiology of the underlying liver disease (Adami et al., 1996; El-Serag et al., 2001; Davila et al., 2005; El-Serag et al., 2006). Given these evident associations, it is imperative for hepatologists and primary care providers to diligently screen for and ensure appropriate management of diabetes in order to proactively prevent the development of liver disease and mitigate risk factors associated with HCC development. In our previous study, the prognostic value of MLR has been demonstrated in a prospective cohort study, showcasing its ability to accurately predict early recurrence and survival after relapse (Wang et al., 2022). In addition, the prognosis of HCC patients following treatment can be predicted by various systemic inflammation parameters, such as the neutrophil-to-lymphocyte ratio (NLR) and platelet-to-lymphocyte ratio (PLR) (Grivennikov et al., 2010; Pinato et al., 2012; Liao et al., 2014). However, unlike the previous studies, our research had a considerably larger and more precise patient population which suggested that higher MLR and WBC level presented a higher risk of mortality in the early HCC patients with DM who underwent liver resection.

Our study revealed a negative correlation between prolonged INR and overall survival in early-stage HCC patients with DM following treatment. INR was introduced to address significant inter-laboratory variability observed in prothrombin time (PT) measurements. Its application extends to standardizing PT values within hepatic disorders, where it has been incorporated into prognostic frameworks such as the Child-Turcotte-Pugh (CTP) score and the model for end-stage liver disease (MELD) (Bonnel et al., 2014). Previous study has proved the INR level was elevated in patients with HCC and comorbid DM (Zhang et al., 2013). In light of the significant involvement of coagulation components in tumor progression, several researchers have investigated the potential of coagulation indicators as prognostic markers for cancer patients. Relevant studies have demonstrated that specific coagulation indicators can serve as predictive biomarkers for prognosis. For instance, PT could be utilized as a prognostic predictor for postoperative recurrence in stage I-III colorectal cancer patients (Ma et al., 2022); abnormally elevated PT levels could function as a straightforward yet effective prognostic indicator for cholangiocarcinoma patients who undergo curative resection (Wang et al., 2019). Moreover, in current study, PT were significantly associated with overall survival in the multivariate analysis. This phenomenon can be attributed to tumor depletion-induced bodily dysfunction, which adversely affects the synthesis of coagulation factors (Wang et al., 2019; Wang et al., 2021; Xiao et al., 2023). The prolonged INR, influenced by the hyperglycemic condition, holds significant clinical implications in HCC patients. It can serve as an additional prognostic indicator for evaluating the outcomes of early-stage HCC patients with diabetes mellitus who have undergone liver resection. Additionally, drinking often leads to impaired liver function, thereby contributing to a poorer prognosis. Moreover, the characteristics of tumor number and size are indicative of high tumor aggressiveness and an unfavorable prognosis for HCC, which is widely acknowledged and requires no further elaboration.

The nomogram has emerged as a dependable clinical tool for quantifying the risk and assessing the prognosis of diverse diseases (Timmers et al., 2013; Kawai et al., 2015; Liang et al., 2015). In our study, the identified prognostic factors were utilized to develop a novel personalized nomogram for the assessment of early HCC patients with DM who underwent liver resection upon admission. The model demonstrated exceptional discrimination and precision in identifying patients at high risk, as evidenced by the outstanding C-index and AUCs achieved in both the internal and external validation cohorts. In addition, we assessed the effectiveness of the nomogram in a subset of individuals with elevated mortality rates. The results indicated that the model exhibited encouraging predictive capability in identifying high-risk early HCC patients with DM who had undergone liver resection. Hence, medical professionals have the opportunity to employ this prognostic model in order to accurately categorize the risk for early-stage HCC patients with DM during their initial assessment.

While the nomogram demonstrated robust predictive performance, it is important to acknowledge certain limitations in our study. Firstly, the retrospective nature of the research inevitably introduced some degree of bias, highlighting the need for future prospective studies to further validate the findings of this nomogram. However, it is noteworthy that internal validation results have confirmed the accuracy and reliability of our developed nomogram; Secondly, our study specifically focused on patients with early-stage HCC and diabetes mellitus (DM) who underwent liver resection. However, it is uncertain whether this nomogram can be applied to patients undergoing transarterial chemoembolization (TACE) or liver transplantation. Therefore, further investigations are necessary to assess the suitability of our nomogram for patients receiving alternative treatments. However, we employed a follow-up duration of up to 8 years in order to construct a precise and reliable nomogram that can effectively provide guidance for clinical practice concerning this specific subset of patients with HCC. Finally, it is imperative to conduct further validation of the short-term mortality risk stratification model in various countries or regions, including the integration of supplementary relevant factors for prospective application.

## Conclusion

In this study, we identified clinical variables such as drinking, tumor numbers, MLR, INR, and WBC at admission as independent prognostic factors for predicting overall survival in early HCC patients after liver resection with DM. We have created and externally validated a predictive nomogram that exhibits excellent discrimination and precision in identifying the subgroup at high risk. This model can be easily utilized by physicians to expedite initial clinical decision-making for early hepatocellular carcinoma (HCC) patients who have undergone liver resection with diabetes mellitus (DM) in routine medical practice.

## Data Availability

The raw data supporting the conclusion of this article will be made available by the authors, without undue reservation.
